# Enhancing Oral Bioavailability of Apigenin Using a Bioactive Self-Nanoemulsifying Drug Delivery System (Bio-SNEDDS): In Vitro, In Vivo and Stability Evaluations

**DOI:** 10.3390/pharmaceutics12080749

**Published:** 2020-08-10

**Authors:** Mohsin Kazi, Abdullah Alhajri, Sultan M. Alshehri, Ehab M. Elzayat, Osaid T. Al Meanazel, Faiyaz Shakeel, Omar Noman, Mohammad A. Altamimi, Fars K. Alanazi

**Affiliations:** 1 Department of Pharmaceutics, College of Pharmacy, King Saud University, Riyadh 11451, Saudi Arabia; abdullah.alhajiri@gmail.com (A.A.); salshehri1@ksu.edu.sa (S.M.A.); fsahmad@ksu.edu.sa (F.S.); maltamimi@ksu.edu.sa (M.A.A.); afars@ksu.edu.sa (F.K.A.); 2 Kayyali Chair for Pharmaceutical Industries, College of Pharmacy, King Saud University, Riyadh 11451, Saudi Arabia; eelzayat1.c@ksu.edu.sa (E.M.E.); otalmeanazel@gmail.com (O.T.A.M.); 3 College of Pharmacy, Almaarefa University, Riyadh 11597, Saudi Arabia; 4 Medicinal Aromatic, and Poisonous Plants Research Center, College of Pharmacy, King Saud University, Riyadh 11451, Saudi Arabia; onoman@ksu.edu.sa

**Keywords:** bioactive self-nanoemulsifying drug delivery system (Bio-SNEDDS), apigenin, solubility improvement, oral bioavailability enhancement

## Abstract

Apigenin (APG) is a very well-known flavonoid for its anti-inflammatory and anticancer properties. The purpose of this study is to improve the solubility and bioavailability of APG using a stable bioactive self-nanoemulsifying drug delivery system (Bio-SNEDDS). APG was incorporated in an oil phase comprising coconut oil fatty acid, Imwitor 988, Transcutol P, and HCO30 to form a Bio-SNEDDS. This preparation was characterized for morphology, particle size, and transmission electron microscopy (TEM). The APG performance was investigated in terms of loading, precipitation, release and stability tests from the optimal Bio-SNEDDS. An antimicrobial test was performed to investigate the activity of the Bio-SNEDDS against the selected strains. Bioavailability of the Bio-SNEDDS was evaluated using Wister rats against the commercial oral product and the pure drug. The results demonstrated the formation of an efficient nanosized (57 nm) Bio-SNEDDS with a drug loading of 12.50 mg/gm which is around 500-fold higher than free APG. TEM analysis revealed the formation of spherical and homogeneous nanodroplets of less than 60 nm. The dissolution rate was faster than the commercial product and was able to maintain 90% APG in gastro intestinal solution for more than 4 h. A stability study demonstrated that the Bio-SNEDDS is stable at a harsh condition. The in vivo pharmacokinetics parameters of the Bio-SNEDDS formulation in comparison to the pure drug showed a significant increase in maximum concentration (C_max_) and area under the curve (AUC _(0–t)_) of 105.05% and 91.32%, respectively. Moreover, the antimicrobial study revealed moderate inhibition in the bacterial growth rate. The APG-Bio-SNEDDS could serve as potential carrier aimed at improving the clinical application of APG.

## 1. Introduction

Low aqueous solubility of drugs has always presented major obstacle towards the development of drug delivery systems, which often compromise drug efficacy and patient compliance [1]. The oral route is considered the most common and convenient route for drug administration. Upon oral administration, the drug is expected to dissolve first and release into the gastrointestinal fluid before the absorption can take place. Poor solubility limits the drug dissolution in the gastrointestinal tract, resulting in low bioavailability that can adversely affect the drug’s therapeutic efficacy [1]. The drugs belonging to the biopharmaceutics classification system (BCS) classes II and IV are particularly facing the challenge of poor solubility or solubility and permeability, respectively. Therefore, they are subjected to extensive investigation for the development of novel effective dosage forms [2].

**Figure 1:**
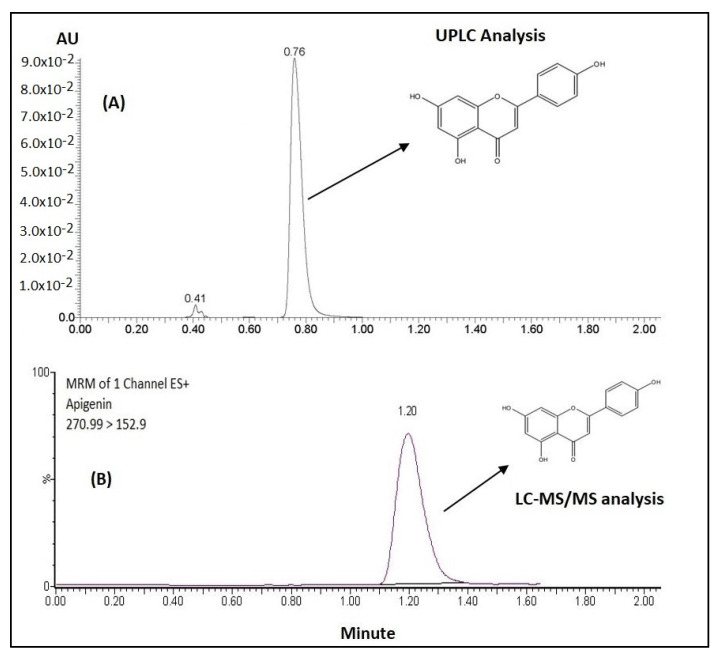
The chemical structure and chromatograms of the apigenin–stable bioactive self-nanoemulsifying drug delivery system (APG-Bio-SNEDDS) by ultra-high-performance liquid chromatography (UPLC) analysis (**A**) and UPLC-MS/MS plasma sample analysis (**B**). (MW: 270.24 g/mol, MP: 345–350 °C).

Apigenin (APG, Figure 1) is one of the most renowned flavonoid from the phenolic compounds with countless nutritional and organoleptic characteristics [3]. It is a plant bioactive compound with various therapeutic activities, such as anti-inflammatory, antioxidant, and antiproliferative activities against neuroblastoma, pancreatic, colorectal, skin and breast cancer cell lines [4,5]. APG produces strong anti-inflammatory effects by reducing the levels of interleukin-6 (IL-6) in animal models. APG also shows antidiabetic properties in a diabetic-animal model [6]. It has growth inhibitory properties in many cancer cells [7]. Furthermore, after absorption into the gastrointestinal tract, APG is able to reach the brain through the circulatory system, where it can cross the blood-brain barrier before exerting its affinity with the GABA_A_-receptor and acting on the central nervous systems. Due to its vast therapeutic activities, APG has attracted the attention of many food and drug manufactures. However, the oral bioavailability of APG is relatively low because of its low aqueous solubility (2.16 μg/mL in water), [2] which has severely limited its further clinical development. Therefore, it is necessary to develop a novel formulation using advance strategies to improve APG oral bioavailability.

APG is a BCS II drug, thus, it might experience low and erratic gastro intestinal tract (GIT) absorption, subsequently decreasing its therapeutic value. Therefore, enhancing the solubility of APG may improve the dissolution rate and hence its oral bioavailability [8,9]. 

To overcome the above-mentioned problem of APG, many conventional techniques have been applied so far using cosolvents, salts, surfactants, cyclodextrins and different polymorphs [10,11,12]. These systems have their own advantages and disadvantages. On the other hand, nanonization is a recent trend which was also utilized in the development of APG as carbon nanopowder [13] and nanocrystals using the supercritical antisolvent process [14,15] and self-microemulsifying systems [16].

It was anticipated that the dissolution of various drugs using nanoemulsion (0–1000 nm particle size) may be able to maintain the drug in a solubilized form in water, which increases the dissolution rate and subsequently enhances absorption of the drug into plasma [17]. In the case of lipid-based self-nanoemulsifying drug delivery systems (SNEDDS), the decreased droplets size increases the rate and extent of absorption and prevents drug degradation in the GIT [18]. 

Potential benefits of SNEDDS technology for poorly soluble drugs are the nanosized particle, increased drug dissolution rate, increased rate and extent of absorption, and reduced variability under fed and fasted states. In addition, it offers various advantages over conventional dosage forms, including the reduction in the dose frequency and possible side effects [19,20]. 

These systems may also protect the drugs which are susceptible to hydrolysis and gastric degradation. Several poorly water soluble drugs have been formulated as self-emulsifying systems to enhance their dissolution and bioavailability, such as cyclosporine A [21] and simvastatin [22]. Within the scope of the current research, the SNEDDS were prepared using bioactive lipid excipients, named the Bio-SNEDDS, which can offer some valuable nutritive and therapeutic effects when compared to the conventional SNEDDS (Table 1) [23,24]. Bioactive oils, for example, black seed oil (BSO), *moringa oleifera* seed oil (MOO), avocado oil (AVO), apricot oil (APO), grape seed oil (GSO), safflower oil (SFO) and coconut oil fatty acid (COFA), were investigated in the Bio-SNEDDS formulation for APG delivery. Several plant bioactive compounds have exhibited functional activities that suggest they could play a remarkable role in preventing a wide range of chronic diseases [25,26]. The Bio-SNEDDS might also improve physical and/or chemical stability of the formulation, the ability to encapsulate them as concentrated volumes, patient compliance/tolerability and a reduction in palatability-related issues. No such studies have been conducted in the past, however, a very limited number of studies have been conducted on the development of APG-loaded conventional SNEDDS that could estimate their bioavailability improvement [27], and the possibility of their use as delivery systems in food and pharmaceutical industries. However, the study explored the in vivo experiment using a suitable animal model. Furthermore, anhydrous SNEDDSs were investigated, which could lead to stabilization of such water-sensitive drugs.

## 2. Materials and Methods

### 2.1. Materials

Model drug APG powder (purity 98.9%) was purchased from Beijing Mesochem Technology Co. Pvt. Ltd. (Beijing, China). The medium-chain mono-, di- and tri-glyceride oils such as Capmul MCM and Imwitor 988 and bioactive oil coconut oil fatty acid (CoFA) were purchased from Abitec corporation, Columbus, OH, USA; Cremer oleo GmbH, Hamburg, Germany. Bioactive oils such as apricot oil (APO), avocado oil (AVO), grape seed oil (GPO), safflower oil (SFO) and non-ionic surfactant PEG 6 sorbitan monooleate (TO106V) and hydrogenated castor oil (HCO30) were obtained from Nikko chemicals co. ltd., Tokyo, Japan. Another two bioactive oils, black seed oil and *moringa oleifera* seed oil (MOO), were extracted by the cold press method (mentioned in the Methods Section). Non-ionic surfactants, such as Tween 80, Tween 85 and co-solvent (e.g., Transcutol P) (TC), were obtained from BASF Germany GmbH, Germany and Gattefosse, France, respectively. Simulated intestinal fluid (SIF) powder, was purchased from biorelevant.com Ltd, London, UK to make fed state intestinal fluid (FeSSIF). All other chemicals that were used in the studies were of analytical grade.

### 2.2. Methods

#### 2.2.1. *Moringa Oleifera* Seed Oil (MOO)

##### Seed Collection and Oil Extraction

*Moringa oleifera* drumsticks from the Moringaceae family (ben oil tree and miracle tree) were allowed to ripe in the tree. The drumsticks were harvested and collected from different areas of Bangladesh. The seeds were separated from pods and seed coats. The amount of seeds obtained per Kg of drumsticks (*Moringa oleifera* fruit) was estimated. The 500 g seeds were dried at a temperature of 70–80 °C in an oven for 6 h and preserved for future use in airtight containers. The collected seeds were crushed by using a manual seed oil press. Seed measurements were taken and the amount of oil extracted per kg of seeds was estimated and preserved for formulation development. 

#### 2.2.2. Black Seed Oil (BSO)

##### Seed Collection and Extraction 

The black seeds were collected from Nigella sativa (N. sativa) and the plant was collected from the southwest part of Bangladesh in March 2019. Approximately 500 g of seeds were taken and cleaned using fresh water. The seeds were then dried under sunlight for at least 48 h. The oil was extracted from the seeds using the cold press technique. Finally, the oil was filtered and transferred to a screw-capped amber-colored glass bottle until further use.

##### BSO Standardization

The main bioactive constituent in the oil of N. sativa is thymoquinone (THQ, chemically known as 2-isopropyl-5-methylbenzo-1,4-quinone) and, hence, it was used for the standardization of BSO. The stock solution of THQ (100 μg/mL) was prepared and used as a reference standard for BSO standardization. Serial dilutions were made from the stock solution of THQ in order to obtain the concentrations of THQ in the range of 0.1–50 μg/mL. The calibration curve of THQ was constructed between the concentration and absorbance. The amount of THQ in BSO was obtained from the calibration curve of THQ. For this, about 1 mL of BSO was separately dissolved in 10 mL of the solvent in volumetric flasks, filtered and utilized for THQ analysis. The amount of THQ in BSO was obtained as 20–50%, which was in accordance with those reported in literature [38,39].

#### 2.2.3. Preparation of the Bio-SNEDDS and Self-Emulsification Assessment

A series of bioactive self-nanoemulsifying formulations were prepared with different ratios of oil, surfactant, co-surfactant and/or cosolvent to achieve the optimal Bio-SNEDDS compositions, which were investigated more closely in terms of their characteristic features and utilization. The optimized formulation was developed in a liquid form and APG was incorporated to maximize drug loading.

APG-loaded formulations were subjected to 1:1000 aqueous dilution in a 20 mL glass beaker, and the contents were gently stirred at ~500 rpm. The formulations were evaluated on the basis of performance indicator tools, such as excipient miscibility, spontaneity and homogeneity/dispersibility. Self-emulsification efficiency was evaluated by following a previously reported [40] visual test.

#### 2.2.4. Particle Size and Polydispersity Index Measurement

The particle size distribution and polydispersity index (PDI) of all the diluted self-emulsifying formulations were measured utilizing a particle size analyzer, Brookhaven (Model 90 plus, Particle Sizing system). The self-emulsifying formulations were diluted at a ratio of 1: 1000 *v*/*v* (formulation: water) and mixed for 1 min before testing [41].

#### 2.2.5. Transmission Electron Microscopy (TEM)

The morphology of the optimized Bio-SNEDDS was investigated using high-resolution transmission electron microscopy Jeol JEM1010 Japan. Each vesicle for TEM measurements was freshly prepared by sample dilution with water (1:1000). Then, 5 μL was deposited on the copper electron microscopy grids standing for 2 min to dry. Then, the surplus was eliminated by absorbing on a filter paper. A drop of osmium was used to stain lipid components. Once dry, it was loaded into the TEM and viewed at 5000–20,000 magnifications.

#### 2.2.6. Equilibrium Solubility Test

Equilibrium solubility of APG in different self-emulsifying formulations was obtained by adopting a reported isothermal method [17]. The excess amount of APG powder was added to the known amounts (1 g) of each formulation and dispersions were vortexed for about 10 min. Each experiment was carried out in triplicates. The obtained samples were incubated at 37 °C in a dry heat incubator until equilibrium was reached. After that, the samples were centrifuged in 1.5 mL Eppendorf tubes at 9800 g for 10 min to exclude excess undissolved drug particles. Then, an aliquot of the supernatant was taken by weight for dilution in appropriate solvent systems before analysis using ultra-high-performance liquid chromatography (UPLC) systems.

#### 2.2.7. UPLC Analysis

The APG content was quantified by injecting samples into a UPLC (Acquity^®^ UPLC, Waters Inc., Bedford, MA, USA) with UV detection at 336 nm. Separation was employed on a reverse-phase isocratic elution using a BEH C18 column (50 mm × 2.1 mm, 1.7 µm) at a flow rate of 0.3 mL/min. The mobile phase comprised a mixture of 0.05 M ammonium formate buffer and 0.1% trifluoroacetic acid (adjusted to pH 2.3) and acetonitrile (72:28, *v*/*v*), run with an injection volume of 1 µL. The column temperature was set at 40 °C. This method was used for APG quantification in all anhydrous formulations and dissolution/stability samples.

#### 2.2.8. APG Precipitation Test

The optimized Bio-SNEDDS was examined and compared with a commercial APG product and pure drug powder to evaluate the APG precipitation rate during aqueous dispersion. Initially, the APG was dissolved in the Bio-SNEDDS at a concentration (10 mg/g) representing 80% of its equilibrium solubility. Then, 1 g of the Bio-SNEDDS and its equivalent APG concentrations of commercial and pure drug powder were diluted in 100 mL water. The dispersion was subsequently agitated and kept in a dry heat incubator at 37 °C for 24 h. During this 24 h dispersion period, 1 mL of the sample was withdrawn at each time point and centrifuged at 9800× *g* for 5 min. An aliquot of the resulting supernatant was taken for dilution with the appropriate solvent prior to assay by UPLC to monitor precipitation. All experiments were carried out in triplicate.

#### 2.2.9. In Vitro Dissolution Test

The dissolution tests were carried out using an automated USP Type II dissolution apparatus (UDT-814, LOGAN Inst. Corp., Doral, FL, USA) with a paddle stirrer at a 100 rpm rotating speed. The weighted amounts of the Bio-SNEDDS (containing 10 mg APG) were filled in “Size 00” HPMC capsules and placed at the bottom of the vessels using a suitable sinker. The dissolution medium contained 900 mL purified water and was maintained at 37 °C temperature. In the case of the “Swanson ultra^®^” capsule (the commercial product), 1% sodium lauryl sulfate was added to maintain the sink condition for quantitation of the drug [42]. Samples of 2 mL each were withdrawn at predetermined time intervals of 5, 10, 15, 20, 30, 60, and 120 min using 10 µm filter tips, and they were replaced with a freshly prepared drug-free dissolution medium immediately. The samples were centrifuged for 5 min at 9800 g; then, the supernatant was carefully taken and analyzed for APG content using the UPLC method discussed above. 

#### 2.2.10. Antimicrobial Activity Test

##### Disc Diffusion Assays

The antimicrobial activity of the optimized Bio-SNEDDS in comparison with commercial APG and various positive controls was evaluated using a disc diffusion method. The antimicrobial evaluation was performed against bacterial and fungal strains. A total of 100 μL of suspensions (containing 10^7^ CFU/mL of bacteria and 10^6^ CFU/mL of yeast) were spread on Mueller-Hinton agar medium and Sabouraud dextrose agar, respectively [43]. Filter paper discs with approximately a 9 mm diameter were prepared and impregnated in 20 μL of each sample (2 mg/disc) and kept on the inoculated Petri dishes. DMSO was used as a negative control and different samples were also dissolved in DMSO. The positive controls for the bacterial strains were ampicillin (2 mg/disc) and kanamycin (2 mg/disc). However, the positive control for the fungal strains was nystatin (2 mg/disc). The obtained Petri dishes were then incubated for 24 h at 37 °C for the bacterial strains and for 48 h at 30 °C for the fungal strains. The antimicrobial efficacy of each sample was determined in terms of the zone of inhibition (mm) against the studied microorganisms, including disc diameter.

#### 2.2.11. In Vivo Oral Bioavailability Study

##### Animals

Healthy male Wistar rats (200–210 g) were procured from the “Central Animal House Facility of the College of Pharmacy, King Saud University (Riyadh, Saudi Arabia)” The animals were kept in plastic cages. Six rats were kept in each cage with a 12 h light/dark cycle at 25 °C ± 2 °C. All experimental procedures were performed in accordance with the “National Institute of Health Guide for the Care and Use of Laboratory Animals (NIH Publications No. 80-23; 1996)” as well as the animal facility guidelines of the “Ethical Committee of Experimental Animal Care Center, College of Pharmacy, King Saud University”. Ethical approval was obtained to carry out these studies (Clearance No. KSU-SE—19-66). The animals were given water ad libitum and fed a standard rat chow diet. All the rats were acclimatized to laboratory conditions for a week before starting the experiments. The rats were divided into three different groups (*n* = 6 in each group) and fasted overnight before administration of the samples.

##### Drug Administration

Prior to oral gavage administration of the animals, pure APG suspension (100 μg mL^−1^) was prepared by homogenously dispersing the required amount of APG in a 0.5% *w*/*v* solution of sodium carboxymethyl cellulose (SCMC) in order to obtain APG suspension. Three different groups were evaluated in the bioavailability study: G1 (pure APG), G2 (APG-Bio-SNEDDS) and G3 (commercial APG capsule). The administered dose of APG of each formulation was 10 mg/kg. Blood samples (approximately 0.5 mL) were withdrawn into a “lithium heparin tube (Improvacuter^®^)” at pre-dose and 1, 2, 4, 6, 12 and 24 h after oral administration of all formulations. Finally, plasma samples were separated from the blood by centrifuging the samples at 50,000 rpm for 20 min. The obtained plasma samples were stored at −80 °C until further use. 

##### Sample Preparation of Apigenin

APG from plasma samples was extracted using a “protein precipitation method”. For this, approximately 100 µL of plasma sample was added to 50 µL of the internal standard (IS; prednisolone 200 µg/mL) and 750 µL of methanol. The obtained mixture was vortexed for about 1 min and centrifuged at 15,000 rpm (15,000× *g*) for about 10 min. The whole supernatant (organic layer) of the sample was transferred into clean centrifuge tubes and evaporated to complete dryness under a stream of nitrogen gas at 45–50 °C. Dry residues were reconstituted with the mobile phase. The supernatant was transferred to a sample vial and 5 µL was injected for quantitative analysis of APG using the UPLC-MS/MS method. Various pharmacokinetic parameters, such as the maximum plasma concentration (C_max_), the time to reach C_max_ (T_max_), the area under curve (AUC_0–24_), the elimination rate constant (Kel) and the relative bioavailability, were determined using “PK Solver Program”.

##### UPLC-MS/MS Plasma Analysis

A validated “UPLC-MS/MS (Waters Acquity, Milford, MA, USA) method” was used for the analysis of APG in rat plasma samples of each formulation. The column used for the analysis of APG was the BEH C_18_ column (50 mm × 2.1 mm, 1.7 µm). The mobile phase was composed of acetonitrile and 0.1% formic acid (35:65, *v*/*v*) which was flowed with a flow rate of 0.25 mL/min. Prednisolone was used as the IS. Detection of the eluted compounds was carried out using ”tandem mass spectrometry using a TQ detector (Waters Corp., Milford, MA, USA) equipped with an electrospray ionization (ESI) source operating in the positive ionization mode. The analysis was carried out with the multiple reactions monitoring (MRM) mode. The selection of ionization pairs (m/z) was as follows: APG: 270.99→152.9 (cone voltage 57 V, collision energy 34 V); IS: 403.172→385.224 (cone voltage 42 V, collision energy 13 V).

##### Pharmacokinetic (PK) Data Analysis

The drug concentrations in the animal plasma at different time intervals were used to analyze its PK profiles by plotting drug concentration-time profile curves. The values of PK parameters were demonstrated as the mean ± standard deviation (SD). The software utilized for the calculation of PK parameters of APG was the Excel add-on PK solver. The non-compartmental PK model was employed to calculate different PK parameters, including the maximum plasma concentration (Cmax), the time to reach the maximum concentration (Tmax), the area under the curve from 0 to t (AUC_0–24_), the elimination rate constant (kz), the half-life (T½) and the mean residence time (MRT).

#### 2.2.12. Stability Test

The optimized Bio-SNEDDS was enrolled in the stability studies according to International Conference on Harmonization (ICH) guidelines. The optimized Bio-SNEDDS of APG was transferred to air-tight amber-colored glass vials in triplicates. The proposed formulation was stored in a climatic stability chamber (KBF-ICH 240/720 series, Binder Gmbh, Tuttlinger, Germany) at 40 ± 2 °C and relative humidity (RH) of 75 ± 5%. Samples were withdrawn at 0, 3 and 6 months at an ambient temperature before further evaluation. The samples were evaluated for drug content percent and changes in physical appearance, such as turbidity, color change or phase separation.

#### 2.2.13. Statistical Analysis

The various pharmacokinetic parameters of the optimized Bio-SNEDDS of APG, the commercial APG formulation and the pure APG were evaluated using a paired t-test using “GraphPad Prism^®^ for Windows (San Diego, CA, USA)”. Statistical significances were assumed when *p* ≤ 0.05, ≤0.001, ≤0.0001. The experiments were carried out in triplicates ± standard deviation.

## 3. Results and Discussion

### 3.1. UPLC and UPLC-MS/MS Analysis of APG

The developed UPLC method, which was used to detect solubilized APG in all anhydrous bioactive lipid formulations, dissolution and stability samples, showed good selectivity. APG was eluted at 0.76 min at a wavelength of 336 nm (Figure 1A). The developed method showed good linearity for APG (r^2^ = 0.9991) over a concentration range of 1.0 and 25.0 µg/mL. The overall analytical data suggested that the APG peak was well separated with precise detection and without any interference; therefore, it was successfully applied to quantify in the Bio-SNEDDS lipid dosage form. 

The method for plasma analysis suggested that the combination of acetonitrile and 0.1% formic acid (35:65, *v*/*v*) as a mobile phase was found to be the most suitable for separating APG (prednisolone was used as the internal standard). The retention time was about 1.2 min for APG, which was eluted without any endogenous interferences from the blank rat plasma. Good linearity (r^2^ > 0.99907) was observed for APG over the range of 5–1000 ng/mL in 0.1 mL of rat plasma (Figure 1B). The overall accuracy of this method was 89.6–105.57% for APG in rat plasma. The lowest quantitation limit for APG was 5 ng/mL in 0.1 mL of rat plasma. The plasma assay results were shown to be consistent, precise and reproducible, and suggested that the methodology can be applied in the assay of the APG in pharmacokinetic studies.

### 3.2. Characterization of the SNEDDS

#### 3.2.1. Bio-SNEDDS Formulation Design

Proper knowledge and selection of the right excipients are important for the successful formulation design in lipid-based systems [21]. In this study, multiple bioactive oils were used with surfactants to develop a series of formulations by a simple technique. Different bioactive oils were mixed together with surfactants and or co-solvents at a constant concentration that represented several types of formulations. Mostly, the formulations were developed in terms of excipient lipophilicity and/or hydrophilicity using pure triglycerides only, mixed mono- and di-glycerides, and nonionic surfactants. The formulations were prepared by simply changing one excipient at a time according to their hydrophilicity (Table 2). In this way, the formulations showed different hydrophilicities, which was the driving force in producing nanoemulsifying formulations. Moreover, the nature of the oils (polarity), and water-soluble surfactants had an effect on the characteristics of the formulation that is more likely to produce a SNEDDS. The polarity of the lipid oils and hydrophilicity of the surfactants were considered to prepare the formulations that were assessed in terms of appearance/droplet size upon aqueous dispersion. 

#### 3.2.2. Assessment of Self-Emulsification Efficiency of the Bio-SNEDDS

The Bio-SNEDDS formulation intended for oral administration can form a fine oil-in-water (*o*/*w*) nanoemulsion upon dilution with an aqueous media, such as water, buffers or GI fluids. The results of self-emulsification evaluation of different Bio-SNEDDSs after dilution with aqueous media (1:100) at an ambient temperature are tabulated in Table 3. The results of the self-emulsification test suggested that only a limited number of bioactive oils were compatible with the studied surfactants. These bio oils were found to give homogeneous, transparent and monodispersed systems with the studied surfactants that formed spontaneously. 

For robust evaluation, a dispersion test was performed for all the Bio-SNEDDSs by diluting them with an aqueous media (1:100). The Bio-SNEDDS were considered as efficient if clear, homogeneous and transparent formulations were obtained after dilution with an aqueous media. Overall, the results of this test suggested that Bio-SNEDDSs F6–F11 were very efficient due to their homogeneity, spontaneity and transparent appearance after dilution with an aqueous media. These formulations were proposed as Bio-SNEDDSs.

Most of the bioactive oils (for example AVO, APO, GPO, SFO MOO in formulations F1–F5) mixed with surfactant T80 yielded poor self-emulsification properties and thus were not considered as Bio-SNEDDSs. They formed immiscible oil globules upon aqueous dispersion, along with hazy/turbid appearances. The formulations F1–F5 containing I988 and T80 were turbid upon aqueous dispersion. However, when these oils were blended with the non-ionic surfactant HCO30, the representative formulations were homogeneous and their appearances were either fine bluish (e.g., F6–F10) or transparent (e.g., F11) upon aqueous dispersion (Table 3). Although F14 produced a transparent appearance, it did not contain any bioactive oils and thus did not qualify as a Bio-SNEDDS. The overall formulation assessment studies suggest that surfactant the HCO30 has higher miscibility with bioactive lipid excipients when compared to other surfactants. This could be due to the matched HLB between the excipients with higher water uptake capacities. Therefore, HCO30 could be a good choice to prepare Bio-SNEDDS formulations.

The formulation assessment during its development suggests that it should be the prerequisite for pharmaceutical formulation design before it moves to further trials. This evaluation can minimize a great deal of error, time and cost of the final product. Therefore, selecting excipients in the formulation design of particular drugs economically and clinically affects significant products in terms of bioavailability and stability.

#### 3.2.3. Formulation Droplet Size and PDIs Analysis

The droplet size analysis of all the formulations showed different particle sizes upon dilution with water. The larger particles were obtained from the dispersion of F1 and F2, which were 2405 and 2640 nm, respectively. In addition, formulations F3–F5 and F12 produced droplet sizes of 987 nm, 821 nm, 234.6 nm and 936.55 nm, respectively. However, the particle sizes for all Bio-SNEDDS formulations were within the range of 57–113.6 nm (Table 3).

It has been reported that the self-emulsifying power of lipid-based formulations such as of the bio-SNEDDS is related with its droplet size distribution [44]. Based on this theory, there are two main proposed criteria to explain the efficiency of such formulations: (a) the rate of self-emulsification and (b) the droplet size distribution of the resultant bio-SNEDDS. The droplet size of these formulations plays a central role in oral absorption of the drug in vivo. The lower droplet size of these formulations results in a larger interfacial surface area, ultimately resulting in enhanced drug absorption and bioavailability. However, it should be considered that the dispersion could be modified substantially in real time by the biological products induced for digestion.

#### 3.2.4. Transmission Electron Microscopy (TEM)

Transmission electron microscopy (TEM) is a very important technique for studying microstructures due to the fact that it produces high-resolution images and it can capture any transition of the structure. Only the optimal nanoemulsifying systems (F11-Bio-SNEDDS) containing no APG were viewed using the TEM, as there was a risk of crystallization of APG with subsequent damage to the TEM.

**Figure 2:**
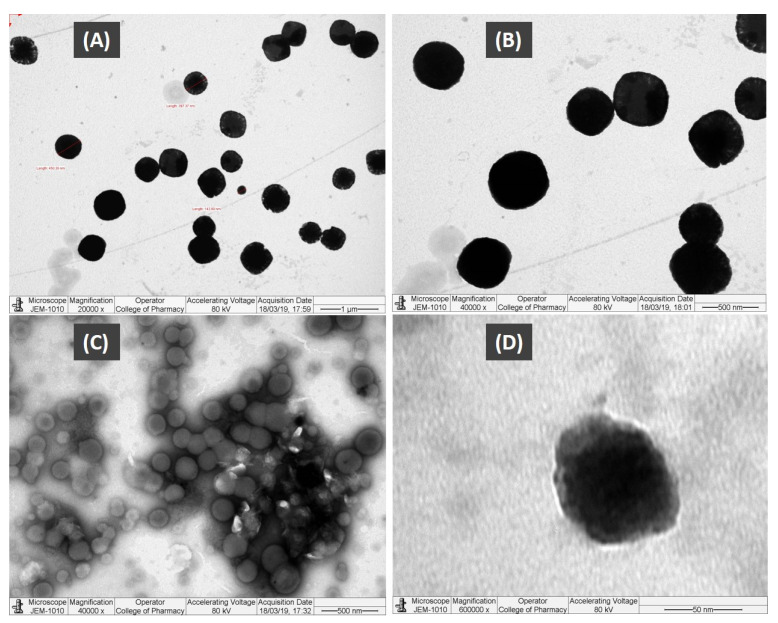
TEM micrographs of the APG*-*loaded Bio-SNEDDS. (**A**–**D**) Images at different magnifications.

The images from the TEM analysis (Figure 2) showed that the droplets of the F11-Bio-SNEDDS have a spherical shape structure and similar sizes, which was confirmed by particle size analysis. The size of the droplets were found to be uniform in the nanoemulsifying systems.

#### 3.2.5. Effect of Oil on the Droplet Size of the Formulation of APG

**Figure 3:**
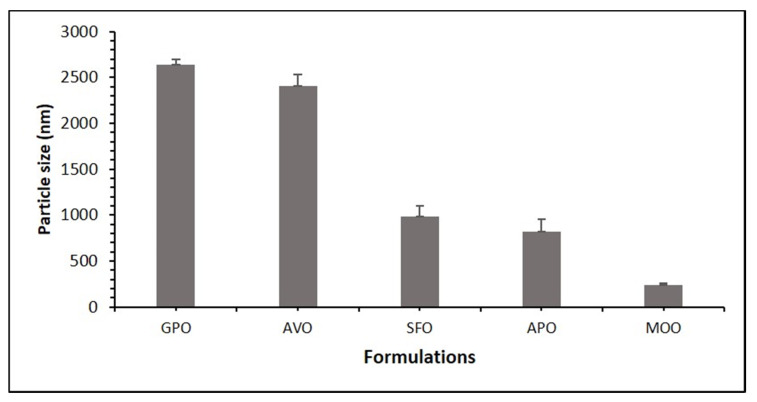
Effect of oils on the droplet size of the APG bioactive formulations. The excipients were mixed as % (*w*/*w*) at a fixed ratio. Bio-SNEDDSs were represented by GPO/I988/T80 (35/15/50)-F2, AVO/I988/T80 (35/15/50)-F1, SFO/I988/T80 (35/15/50)-F3, APO/I988/T80 (35/15/50)-F4, and MOO/I988/T80 (35/15/50)-F5, respectively.

The particle size analysis of these formulations (F1–F5) was set randomly to assess if using different types of bioactive oils will affect the particle size or not. The same amount of I988 and T80 was used when changing the oil. F2 (grape seed oil) gave a high particle size, and F4 (moringa oleiferra oil) gave the lowest particle size, which indicates that using moringa oleiferra oil is a good candidate in creating a formulation that is intended for reducing particle size, as shown in Figure 3. In our past studies, it was found that optically transparent bio-SNEDDSs with a reduced droplet size could be formed by controlling the surfactant-to-oil ratio, the oil composition (free fatty acids + salts) and their properties [26]. The bioactive oils (GPO, AVO, SFO, APO and MOO) used in the studies have unsaturated fatty acids with considerable impact on medicine and nutrition. Among them, SFO and MOO are more water soluble (hydrophilic lipophilic balance (HLB) ≈ 8, contained more linoleic acid) than GPO, AVO and APO (HLB ≈ 7, contained more oleic acid). In addition, water uptake rate is lower for GPO and AVO when compared to other oils, thus making them less polar/compatible with T80 and poorly dispersed in aqueous media. In aqueous systems, polar lipids spontaneously form nanoparticles with uniformity among oil droplet sizes and better stability [45].

#### 3.2.6. Effect of the Surfactants on the Droplet Size of Formulation of APG

**Figure 4:**
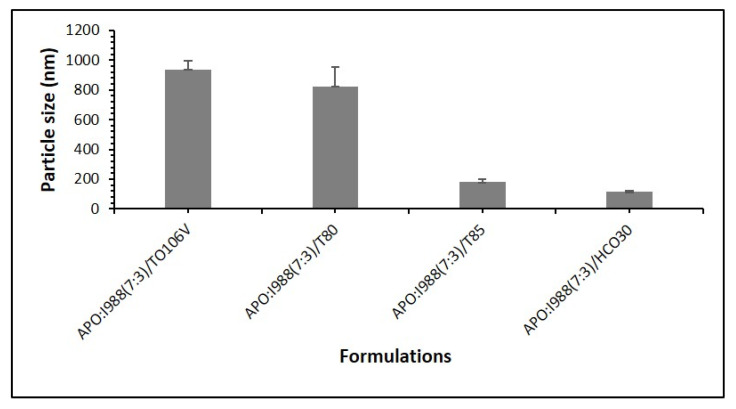
Effect of surfactants on the droplet size of the APG bioactive formulations. The excipients were mixed as % (*w*/*w*) at a fixed ratio. Bio-SNEDDSs were represented by APO/I988/TO106V (35/15/50)-F12, APO/I988/T80 (35/15/50)-F4, APO/I988/T85 (35/15/50)-F13, and APO/I988/HCO30 (35/15/50)-F6, respectively.

Particle size analysis of these formulations (F4, F6, F12 and F13) was set to assess if using different types of surfactants will affect the particle size or not. The same ratio of APO, I988 and surfactants was used with changing surfactant types. As shown in Figure 4, the formulation F12 (with TO106V) formed the highest particle size and F6 (with HCO30) formed the lowest particle size (decreased ≈ 7-fold). The data indicate that HCO30 could be a good candidate in a formulation that is intended for reducing particle size, as shown in Figure 4. The fixed oil combinations in the Bio-SNEDDS were stabilized with different nonionic surfactants. Each surfactant acted differently at the oil-water interface of the Bio-SNEDDS according to their molecular structure, which determines their HLB and ability to adsorb to the oil-water interface with reduced interfacial tension. The higher the HLB value, the higher the solubility of the surfactant in water and the more likely it is to reduce the droplet size of the formulation. Although the HLB value of T80 is higher (HLB-15), the particle size was not reduced due to less effectiveness in migration to the interface when compared to T85 (HLB-11) and HCO-30 (HLB-11). This could be due to the surfactant compatibility (charge and its interaction with the oil phase) with the APO mix. Moreover, the homogenization of different phases in the system and their viscosity can impact the droplet size of the Bio-SNEDDS upon aqueous dispersion [26].

#### 3.2.7. APG Solubility in Lipid Formulation

Solubility is an important part of any drug formulation because it offers the required information regarding the maximum dose that can be included in a single unit dosage form. All these formulations were tested for maximum equilibrium solubility by being maintained at 37 °C temperature for 3 days. Solubility of APG in various lipid-based formulations is presented in (Table 3). The data from the study showed that most of the lipid-based Bio-SNEDDS were not able to achieve the solubility even 3 mg/g, except F11 and F14 of 12.50 mg/g and 19.02 mg/g, respectively. This suggests that APG may prefer hydrophilic substances in the formulation.

#### 3.2.8. The Best Optimized Formulation

**Figure 5:**
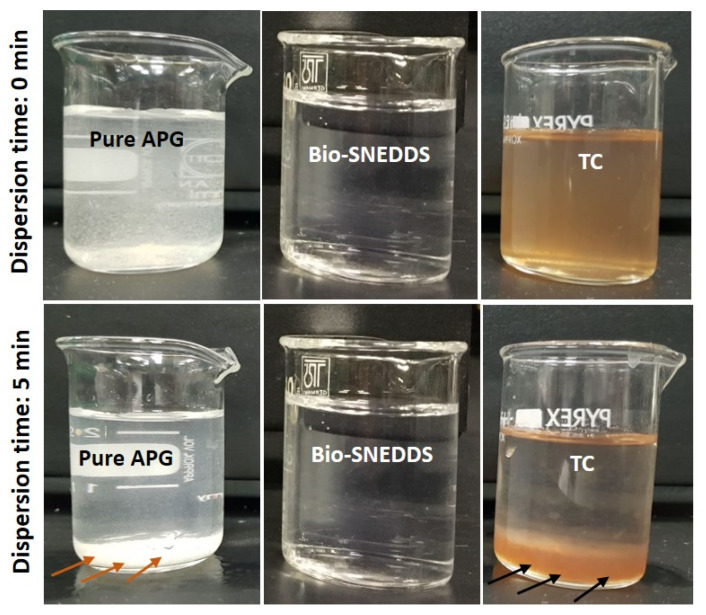
Appearance of pure APG powder and the representative formulations upon aqueous dilution with water after the initial and 5 min dispersion time. Arrows show the sedimentation/precipitation of APG after aqueous dispersion.

The selection criteria for the optimized formulation were higher solubility among other formulations, low droplet size, better dispersibility, and transparency in appearance. When considering all the above determining parameters, it was shown that F11-Bio-SNEDDSs had the best possible balanced characteristics to be selected as the optimized formulation. From the overall solubility, droplet size and physical assessment data, CoFA with a water-soluble cosolvent and surfactant yielded the best nanoemulsifying systems (SNEDDS) with a droplet size of 57 nm and a high solubility of 12.50 mg/g. Its appearance was transparent upon aqueous dilution and no precipitation was noticed when compared with pure APG powder and formulation F14 (Figure 5). This Bio-SNEDDS formulation was efficient in formulating APG and was selected for further studies, such as dynamic dispersion, stability, antimicrobial and pharmacokinetic evaluations.

### 3.3. Dynamic Dispersion Studies

In vitro dispersion is a required experiment to evaluate the capability of lipid-based vehicles to carry drugs upon aqueous dispersion through the intestine. It is always challenging for the drug to be in a solubilized state when it partitions from the vehicle into the aqueous medium. The test is appropriate for the prediction of whether drug precipitation is likely to occur prior to digestion. The main objective of the in vitro dispersion test is to select the appropriate formulation for further in vivo studies. An appropriate formulation maintains the drug in the solution before it reaches the systemic circulation. A range of biorelevant dissolution test media and experimental methodologies have been developed by Dressman and colleagues that have an established application in drug release studies of lipid-based oral formulations.

Dispersion evaluation could be performed using a standard dissolution apparatus, but considering that the drug is initially in the solution form in the anhydrous bio-SNEDDS, the emphasis must be placed on detecting unusual drug precipitation during GI transit rather than dissolution. The avoidance of drug precipitation upon the dispersion process is the main goal of pharmaceutical applications of bio-SNEDDSs. Drug precipitation can be avoided by increasing the solubilization potential of the bio-SNEDDS extensively over the desired drug concentration.

**Figure 6:**
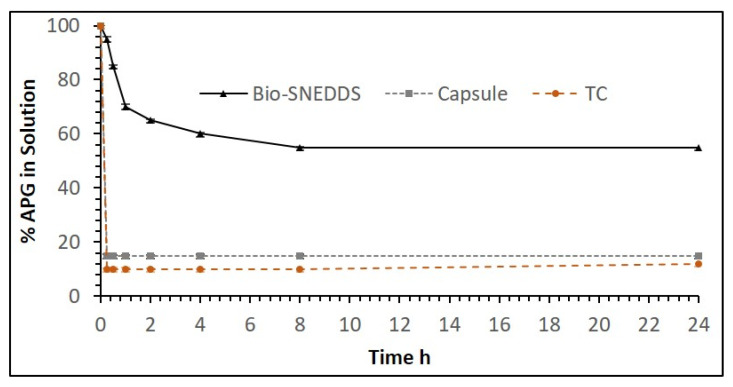
The percentage of APG in the solution over 24 h after 1:100 dilution in the aqueous media (water). Formulations studied: the Bio-SNEDDS of F11 (CoFA: CMCM: TC (3:1:1)/HCO30 [1/1]), the commercial APG capsule and F14 (TC only formulation). Data are presented as mean ± SD, (*n* = 3).

**Figure 7:**
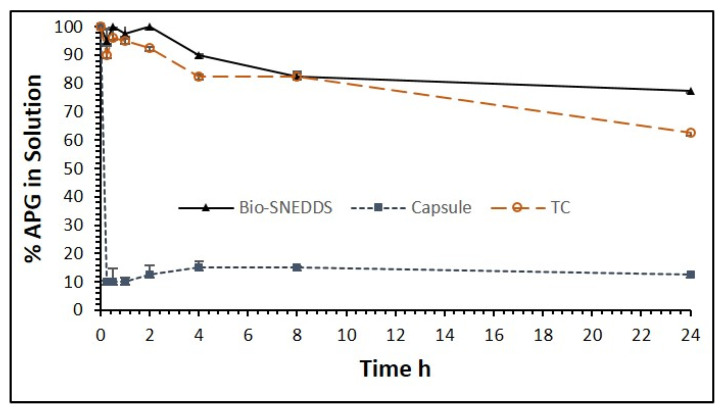
The percentage of apigenin in the solution over 24 h after 1:100 dilution in the aqueous media (fed state intestinal fluid—FeSSIF). Formulations studied: the Bio-SNEDDS F11 (CoFA: CMCM: TC (3:1:1)/HCO30 [1/1]), the commercial APG capsule and F14 (TC only formulation). Data are presented as mean ± SD, (*n* = 3).

The APG precipitation experiment was carried over 24 h out in aqueous media (water) and fed state simulated intestinal fluid (FeSSIF) for the optimum Bio-SNEDDS formulation F11 in comparison with the commercial APG product and F14 (Transcutol P, the extremely water-soluble formulation). The results from the aqueous media (water) shown in Figure 6 depict that formulation F11 maintained more than 85% of the drug in the solution within 1 h, and the commercial product and pure APG kept 85% and 90% of the drug out of the solution in the same hour, respectively. On the other hand, the F11-Bio-SNEDDS maintained more than 95% of the drug in the solubilized state, while commercial APG maintained only around 20% of the drug in the FeSSIF media (Figure 7). However, pure APG was maintained by 70% in the FeSSIF media, which is assumed to be due to the bile salt phospholipid contents present in the media. The overall dispersion studies confirmed that the Bio-SNEDDS (mixed glyceride contents) can retain a good amount of the drug in the solution for 4 h up to 24 h in the intestinal media. Thus, the bioavailability of APG can be significantly increased by this Bio-SNEDDS as the APG remains in the solubilized form during the digestion time (at least a 4 h period) in vivo.

### 3.4. In Vitro Release Studies

**Figure 8:**
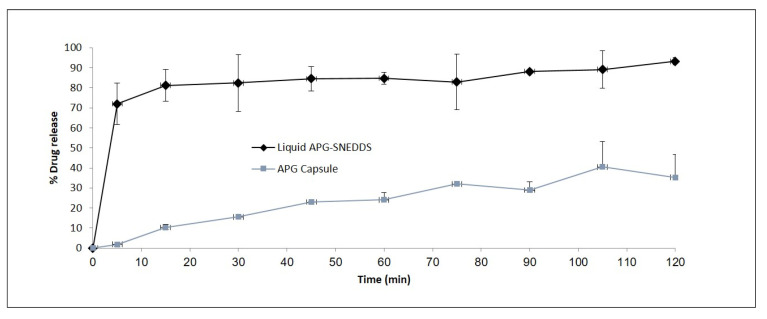
In vitro dissolution profiles of the representative APG-loaded liquid F11-Bio-SNEDDS (CoFA:CMCM:TC (3:1:1)/HCO30 [1/1]) the and commercial APG capsule in aqueous media (pH 6.8) over 120 min. Data are presented as mean ± SD, (*n* = 3).

In vitro release studies were conducted for APG to obtain insight into the release behavior of the drug from the Bio-SNEDDS as well as from the marketed product. The drug-release profile of the commercial APG capsule and the APG-loaded Bio-SNEDDS (F11) is provided in Figure 8. An immediate release was noted with the Bio-SNEDDS. The quick release of the APG after the first 5 min of the experiment from the F11-Bio-SNEDDS was 73.12%, which was perhaps due to the nanosized particles of the larger surface area of the liquid immediate release formulation. The APG-loaded Bio-SNEDDS showed a cumulative release of 89.2% of the drug in comparison to the APG capsule with 31.2% 120 min after the study had commenced. To understand the mechanism of release of the drug from the Bio-SNEDDS, the release data of F11 and commercial product were compared with a previously conducted dynamic dispersion test in FeSSIF media (Figure 7).

Bio-active excipients can be incorporated in the liquid SNEDDS formulation to assist in the quicker dissolution process of the drug, as proved by the release study here. In addition, specialized dosage forms (for example solid dosage form) can be formulated that improve the dissolution rate through various mechanisms.

#### Antimicrobial Activity (Disc Diffusion Assays)

It was anticipated that CoFA has proteins which can adsorb bacteria and later on kill them. Therefore, the formulation developed with CoFA will have a sterilizing capacity or will be protected from any microbial/bacterial growth during the storage conditions as well as in application. The representative Bio-SNEDDS showed a moderate bactericidal activity against Gram-positive and Gram-negative bacteria, and a strong antifungal activity against *Candida albicans* (Table 4). Commercial apigenin capsule did not show any activity against Gram-positive or Gram-negative bacteria. However, it showed slight antifungal activity against *C. albicans.* No inhibition was observed with the solvent control (DMSO) that was used as a solvent. Bacterial and fungal growth was inhibited by the antibiotics and was used as control. Ampicillin inhibition zones varied from 25 mm for *Staphylococcus Aureus* to 27 mm for *Escherichia coli*; kanamycin inhibition zones ranged between 23 mm for *S. Aureus* and 25 mm for *E. coli;* and the nystatin inhibition zone was 25 mm for *C. albicans*.

### 3.5. Stability of Apigenin in the SNEDDS

The optimized Bio-SNEDDS formulation (F11) was studied to investigate the effect of temperature and time on a storage period of up to six months. The purpose here was to prove that the optimized formulation F11 in the liquid form could be stable up to 3 months in drastic conditions with no significant change in particles size, zeta potential or poly dispersity. Table 5 represents the appearance, particle size, zeta potential value and drug content of the formulation at different timelines. The data in Table 5 show a decrease in drug content with some changes in appearance from the three to six month period. Furthermore, an increase in particle size was observed as time passed, which is usual when a liquid formulation is kept at drastic conditions for a long time. However, the overall results were within the acceptance criteria, except for particle size, whose appearance became somewhat hazy after six months. It is worth mentioning that the acceptance criteria to be selected for the Bio-SNEDDS should be a particle size of less than 200nm, a transparent appearance and enough drug loading to meet the single unit dosage requirement. To further prove its stability, dissolution studies may be recommended to examine APG release in the future. In another way, solidification of lipid nanoformulation could help stabilize the dosage form for a longer period than in its liquid form [46]. Our previous article with fenofibrate solid SNEDDSs showed the improved stability when an inorganic adsorbent was used as solidified materials [47].

### 3.6. Pharmacokinetic Study

**Figure 9:**
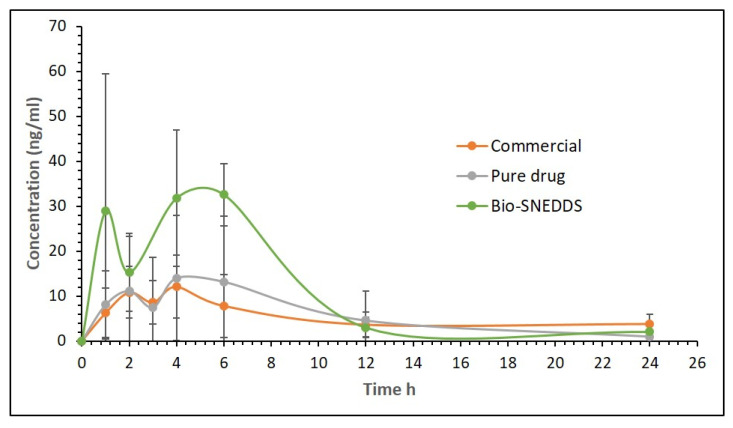
Plasma concentration–time profiles of APG after a single oral administration of the Bio-SNEDDS formulation [F11, CoFA:CMCM:TC (3:1:1)/HCO30 [1/1]] and APG pure powder to rats at a dose equivalent to 10 mg/kg (Mean ± SD, *n* = 6).

The oral pharmacokinetic (pk) analysis of APG was performed on rats to establish the relevance of the newly developed Bio-SNEDDS in actual biological matrices. The pk profile of an optimized Bio-SNEDDS (F11) was evaluated to analyze APG in rat plasma after oral administration. The main objective of this study was to correlate the solubility of APG with the enhancement of its oral bioavailability. The pk profile of an optimized Bio-SNEDDS in comparison with pure APG suggested an enhancement in relative bioavailability of APG. APG was absorbed after oral administration with peak plasma levels attained in 1.1 h and 3.6 h. The C_max_ of oral administration of APG was found to be 21.38 ± 14.35 ng mL^−1^ and the T_max_ of 3.6 was obtained, while the oral administration SNEDDS formulation of APG C_max_ and T_max_ were 43.84 ± 20.13 ng mL^−1^ and 4.2 h, respectively (shown in Figure 9). When APG transported through the GIT, a fraction of the drug was absorbed into the blood, and the remaining amount was effluxed back to the luminal side of the intestine and reabsorbed; thus, it is clear with such a bimodal concentration time profile. However, this may not only be because it is triggered by enterohepatic circulation, but could also be due to partial gastric digestion. The bimodal profile in this study was characterized by a rapid initial release of the drug from the lipid-based Bio-SNEDDS followed by a constant release rate; then, a second mode of fast drug release at the terminal phase. The APG-release profile was assumed to be selectively modified by the lipid/surfactant viscosity, the concentration, and their combination. Previously, bimodal release profiles were obtained for aspirin, ibuprofen, adinazolam, flurbiprofen, and several other drugs [48]. The C_max_ value of APG from the representative Bio-SNEDDS formulation was significantly increased by two-fold (*p* < 0.0001). The AUC _0–t o_ of APG also significantly increased in the SNEDDS-treated group as compare to the APG only-treated group by 91.32% (from 146.54 ± 139.63 to 280.37 ± 58.62, Table 6). The improvement in bioavailability of APG of the Bio-SNEDDS (F11) formulation may be due to the decreased particle size and increased solubility of APG. The increase in relative bioavailability was found to be 2.04-fold. The calculated oral clearance decreased by 71.28% (CL/F from 0.1195 ± 0.09432 to 0.0343 ± 0.0057 mL h^−1^; *p* < 0.0001). The bioavailability study in rats shows that it is possible to improve the bioavailability of APG if given in the Bio-SNEDDS formulation.

There are several possible mechanisms which could increase the bioavailability of Bio-SNEDDSs. The faster uptake of drug-loaded formulations from the resultant emulsion at the absorption site initiated rapid onset of action from the Bio-SNEDDS. High drug solubilization capacity and the self-emulsifying ability of the F11 formulation may have contributed to the increased AUC.

## 4. Conclusions

The representative Bio-SNEDDS formulated for APG in the current study provide collective advantages, such as superior self-emulsification efficiency with improved physical stability, high drug loading capacity, antibacterial activity, and elevated APG bioavailability. The most appropriate bioactive excipient that is able to produce an efficient Bio-SNEDDS was coconut oil fatty acid. This work reveals the importance of Bio-SNEDDS development for APG, which may result in a synergistic benefit in treatment along with a lower dose administration. Based on the in vitro and in vivo evidence reported here, APG, a natural bioactive flavone-type molecule, could play a key role in the prevention and treatment of emerging global health issues. However, there is a need for further research to investigate the applicability and synergistic effect of multifunctional bioactive excipients to target disease for a wide variety of poorly soluble APIs.

## Figures and Tables

**Table 1:** The major chemical compositions and therapeutic benefits of bioactive excipients used in current studies, along with their suppliers’ information.

Name	Chemical Description	Bioactive Characteristics	Supplier
Coconut oil fatty acid (CoFA)	CoFA has a higher content of tocotrienols and tocopherols (forms of vitamin E), sterols (precursors to fat-soluble vitamins and steroid hormones). It has proteins which can adsorb and kill bacteria.	Antioxidant activity; antimicrobial effects [28,29].	Cremer Oleo GmbH & Co, Hamburg, Germany
Avocado oil (AVO)	AVO is high in monounsaturated fatty acids, particularly oleic acid, followed by palmitic and linoleic acids. Avocado oil is naturally low in acidity, has the highest smoke point and contains more than 50% monounsaturated fat, which makes it less prone to oxidation.	Antioxidant activity; promotes the accumulation of HDL cholesterol; anti-inflammatory activity [30].	Nikkol chemical co, Tokyo, Japan
Grape seed oil (GSO)	GSO has a high content of polyunsaturated fatty acids, such as omega-6 and omega 9, in the range of 85–90%. It has a large amount of phenolic compounds, including flavonoids, carotenoids, phenolic acids.	Chemo preventive to control cancer; anti-inflammatory; anticholesterol agent; an immunostimulating effect [31,32].	Nikkol chemical co, Tokyo, Japan
Apricot oil (APO)	APO contains various phytochemicals, such as pro-vitamin A beta-carotene; polyphenols, including catechins and chlorogenic acid; as well as oleic acid and linoleic acid (omega-9).	Antioxidant activity; anti-inflammatory and respiratory support; anticancer potential and antibacterial [33].	Nikkol chemical co, Tokyo, Japan
*Moringa Oleifera* (MOO)	MOO is rich in oleic acid, tocopherols and sterols. It has proteins which can adsorb and kill bacteria, as well as a high content of β-carotene and vitamin C.	Antioxidant; anti-inflammation; remedy for infectious diseases, hematological diseases, hepatorenal diseases, gastrointestinal disorders, rheumatic arthritis, and cardiovascular diseases [34].	Cold pressed, Dhaka, Bangladesh
Black seed oil	Thymoquinone 20–50%	Anticancer activity; anti-infective; anthelmintic; antimicrobial; anti-viral, antibacterial, antifungal, antiparasitic and antispasmodic activity; anti-inflammatory; antihistaminic and analgesic; antipyretic, and antiulcerative activity [35,36].	Cold pressed, Dhaka, Bangladesh
Safflower oil	Rich in linoleic acid and omega-6 fatty acid. The seeds contain a lignan glycoside known as tracheloside; serotonin derivatives.	Capable of protecting against heart disease; antioxidant; anti-inflammatory and respiratory support [37].	Nikkol chemical co, Japan
Apigenin	Flavonoid, phytopolyphenol.	Anti-inflammatory; antioxidant; antistress; antidiabetic, induces autophagy (cellular waste-recycling system) in leukemia cells; antibacterial agent [5].	Beijing Mesochem Technology Co. Ltd., Beijing, China

**Table 2:** Formulation composition using various fruit oils, medium- and long-chain mixed glycerides, and non-ionic surfactants. The excipients are mixed as % (*w*/*w*).

F. N.	Bioactive Oil *							Mono- and Di-Glycerides Oil **		Nonionic Surfactant and Co-Solvent ***				
	AVO	GPO	APO	MOO	SFO	BSO	CoFA	CMCM	I988	TO106V	HCO30	T85	T80	TC
F1	35								15				50	
F2		35							15				50	
F3					35				15				50	
F4			35						15				50	
F5				35					15				50	
F6			35						15		50			
F7	35								15		50			
F8						35			15		50			
F9						35		15			50			
F10			35					15			50			
F11							30	10			50			10
F12			35						15	50				
F13			35						15			50		
F14														100

* Avocado oil (AVO), grape seed oil (GPO), apricot oil (APO), moringa seed oil (MOO), safflower oil (SFO), black seed oil (BSO), and coconut oil fatty acid (COFA). ** Capmul MCM and Imwitor 988. *** Surfactant, PEG 6 sorbitan monooleate (TO106V), hydrogenated castor oil (HCO30), Tween 80, Tween 85 and co-solvent Transcutol P (TC). F.N denotes formulation number.

**Table 3:** Appearance of various formulations and spontaneity upon aqueous dispersion. Additionally, composition of samples, concentration (%) of excipients in anhydrous formulation, droplet size, polydispersity index (PDI) and equilibrium solubility of various formulations of APG. ND denotes “not detected”.

No	Formulation (*w*/*w*)	Appearance	Mean Droplet Size (nm)	PDI	Solubility (mg/g)	Bio-SNEDDS
**F1**	AVO:I988(7:3)/T80[1/1]	Turbid (Coarse)	2405 ± 124	0.194	1.05 ± 0.32	No
**F2**	GPO:I988(7:3)/T80[1/1]	Turbid (Coarse)	2640 ± 59	0.163	ND	No
**F3**	SFO:I988(7:3)/T80[1/1]	Turbid (Coarse)	987 ± 112	0.781	0.39 ± 0.01	No
**F4**	APO:I988(7:3)/T80[1/1]	Turbid (Coarse)	821 ± 135	0.366	1.20 ± 0.32	No
**F5**	MOO:I988(7:3)/T80[1/1]	Bluish (Coarse)	234.6 ± 24.4	0.470	2.04 ± 0.19	No
**F6**	APO:I988(7:3)/HCO30[1/1]	Bluish (Fine)	113.6 ± 5.8	0.476	2.86 ± 0.09	Yes
**F7**	AVO:I988(7:3)/HCO30[1/1]	Bluish (Fine)	104.5 ± 6.3	0.579	1.90 ± 0.21	Yes
**F8**	BSO:I988(7:3)/HCO30[1/1]	Bluish (Fine)	104.70 ± 21.35	0.873	1.53 ± 0.05	Yes
**F9**	BSO:CMCM(7:3)/HCO30[1/1]	Bluish (Fine)	97.86 ± 11.25	0.636	1.66 ± 0.02	Yes
**F10**	APO:CMCM(7:3)/HCO30[1/1]	Bluish (Fine)	62.85 ± 4.68	0.407	2.42 ± 0.03	Yes
**F11**	CoFA:CMCM:TC (3:1:1)/HCO30 [1/1]	Transparent	57.00 ± 14.80	0.419	12.50 ± 0.24	Yes
**F12**	APO:I988(7:3)/TO106V [1/1]	Turbid	936.55 ± 58.23	0.888	ND	No
**F13**	APO:I988(7:3)/T85[1/1]	Turbid (Fine)	179.10 ± 22.75	0.423	ND	No
**F14**	TC	Transparent	21.34 ± 3.13	0.356	19.02 ± 1.02	No

**Table 4:** Antimicrobial activities of the representative F11-Bio-SNEDDS and the APG commercial capsule against *Staphylococcus Aureus*, *Escherichia coli* and *Candida albicans*.

Sample	Zone of Inhibition against *S. Aureus* (*ATCC25923*)/mm	Zone of Inhibition Against *E. coli* (*ATCC25922*)/mm	Zone of Inhibition against *C. Albicans* (*ATCC60193)*/mm
Bio-SNEDDS	12	9	18
Commercial APG	-	-	9
Ampicillin	25	27	NT *
Kanamycin	23	25	NT *
Nystatin	NT *	NT	25
DMSO	-	-	-

NT * not tested.

**Table 5:** Time-dependent stability studies showing the drug recovery after three and six months at 40 °C and 75% relative humidity (RH). An amount of 10 mg of APG, which was 80% of the equilibrium solubility of the Bio-SNEDDS formulation F11-CoFA: CMCM: TC (3:1:1)/HCO30 [1/1], was kept in the incubator for the stability test.

Time (Months)	Drug Content (%)	Particle Size (nm)	Zeta Potential (mV)	Bio-SNEDDS Appearance
0	100	57.12 ± 11.45	−14.21 ± 3.12	Transparent
3	96.8	79.20 ± 8.23	−13.90 ± 2.11	Transparent
6	90.8	269.93 ± 13.85	−16.50 ± 1.23	Hazy

**Table 6:** Pharmacokinetic parameters of pure APG powder vs. the APG-loaded Bio-SNEDDS (formulation used: F11-CoFA:CMCM:TC (3:1:1)/HCO30 [1/1]).

Pharmacokinetic Parameters	Pure APG (Mean ± SEM, *n* = 6)	APG Bio-SNEDDS (Mean ± SEM, *n* = 6)
T_max_ (h)	3.60 ± 1.67	4.20 ± 2.04
C_max_ (ng/mL)	21.38 ± 14.35	43.84 ± 20.13
AUC_0–t_ (ng *h/mL)	146.54 ± 139.62 *	280.37 ± 58.62 *
AUC_0–∞_ (ng *h/mL)	157.59 ± 136.13	298.77 ± 55.72
CL/F ((mg)/(ng/mL)/h)	0.1195 ± 0.0943 ***	0.0343 ± 0.0057 ***
T_1/2_ (h)	7.87 ± 5.03	4.77 ± 2.26

* Data are expressed as mean ± SD (*n* = 6), * *p* < 0.05, and *** *p* < 0.0001. T_max_ = time of peak concentration, C_max_ = peak of maximum concentration, AUC0→t = area under the concentration time profile curve of up to 24 h, AUC0→∞ = area under the concentration time profile curve extrapolated to infinity, T1/2 = half-life, CL/F = oral clearance.
